# Cost-effectiveness of molar single-implant versus fixed dental prosthesis

**DOI:** 10.1186/s12903-018-0604-5

**Published:** 2018-08-20

**Authors:** Arai Korenori, Kawakami Koji, Teranishi Yuki, Tatsunori Murata, Tanaka-Mizuno Sachiko, Baba Shunsuke

**Affiliations:** 1 Department of Oral Implantology, Osaka Dental University, 8-1, Kuzuhahanazonocho, Hirakata, Osaka, Japan; 2 Department of Pharmacoepidemiology, Graduate School of Medicine and Public Health, Kyoto University, Kyoto, Japan; 3 Department of Biotatistics, Shiga University of Medical Science, Otsu, Japan

**Keywords:** Dental implant, Cost-effectiveness analysis, Economic evaluative, Markov model, Patient reported outcome

## Abstract

**Background:**

This study evaluates the cost-effectiveness of implants (Implant), insurance fixed dental prosthesis (IFDP) and private fixed dental prosthesis (PFDP) for a single intermediate missing tooth in the molar region to calculate the Incremental Cost Effectiveness Ratio (ICER).

**Methods:**

The Markov model for cost-effectiveness analysis of the Implant, IFDP and PFDP was carried over maximum 30 years. The starting age for prosthetic treatment was decided to be 50 years. The General Oral Health Assessment Index (GOHAI) was used for the indicator of effectiveness as an oral health QOL value. The GOHAI value was collected from patients who visited the Department of Oral Implantology of Osaka Dental University between September 2014 and March 2016. In addition, the Tornado diagram was drawn and Monte-Carlo simulations made for sensitivity analysis.

**Results:**

From the analysis of survey of QOL of each stage and treatment, the selection of an Implant led to a higher QOL value than FDP. However, the estimated 30-year cost for IFDP was lower than Implant. It also became evident that PFDP had an extended dominated condition compared with IFDP and Implants. The ICER on the Implant versus IFDP was €1423.00.

**Conclusions:**

These results suggest that a better of QOL value can be obtained from an Implant than from IFDP or PFDP. An evaluation form using an indexed scale for oral health-related aspects needs to be developed that is also consistent as an indicator of effect.

## Background

The value of medical technologies has been questioned with the advent of the global revolution in medical care in recent years. Just like other countries, concerns about the failure of the market mechanism for medical care and the sustainability of universal health insurance coverage have increased even in Japan. An increase in the burden of pain and reduction in benefits are said to be inevitable; thus, the ‘value’ of medical technology is being questioned with the incorporation of Health Technology Assessments (HTAs) [1]. HTAs represent an interdisciplinary research area that investigates the impact of medical technologies on health from economic, systematic, social and ethical perspectives while considering possible options. The purpose of the HTA is to provide information for formulating patient-based, safe and effective medical care policies aimed at achieving the very best value. The academic basis for this is pharmacoeconomics. This discipline evaluates the medical impact for a patient through the adaptation of medical technology and the comparative consideration of the required cost. This is essentially a quantification of the relative value of medical technologies and the science of examination of the cash value of medical care [2]. Therefore, the main objective of the economic evaluation is not to only reduce the cost of medical care but also adopt a fair approach from the perspective of value-based public policy, which facilitates the fair evaluation of innovations in medical care [3]. There has been much pharmacoeconomics research in medical science but little in the area of dentistry. Thus, this study focuses on the cost of dental care in Japan. Examining the composition of health care renumeration points per day by care type, prosthetic treatment accounted for a higher proportion than other treatments according to reports by the Ministry of Health, Labour and Welfare in Japan [4]. Therefore, this study undertakes an economic evaluation of prosthetic treatment for intermediate loss of molars. Implants do not invade neighboring teeth. It is an important treatment option in contemporary dental medicine from the viewpoint of “Minimal Intervention” [5]. In the Japanese public medical care insurance, only the FDP by metals that include gold–silver–palladium or the RPD can be selected for prosthetic treatment of a missing unilateral lower jaw first molar. Therefore, the FDP by hybrid ceramics or porcelain crowns and the implant treatment will be completely self-funded, and medical treatments are not covered by public insurance. So the prosthetic treatment for the intermediate loss of molars involves treatment that mainly uses insurance fixed dental prosthesis (IFDP) (materials: metals that include gold–silver–palladium). Options also include Implants other than insurance adaptation and private fixed dental prosthesis (PFDP) (materials: hybrid ceramics and porcelain crowns). There are health economic assessments concerning Implants [5]; in previous studies analysing cost-effectiveness using the original survey form, Implants were seen as a dominant strategy compared with fixed dental prosthesis (FDP) [6]. In addition, in prior studies in South Korea where the endpoint is taken to be the survival rate of the prosthetic device, a 20% reduction in the cost of Implant treatment would result in Implant being more dominant than FDP [7]. However, to date, there has been little economic assessment research using the measurement of oral quality of life (QOL), which is widely used worldwide in relation to the effect. This study thus aims to undertake a cost effectiveness analysis (CEA) of the prosthetic treatment for the intermediate loss of a single molar to compare Implant and FDP from cost and QOL perspectives.

## Methods

### Methods-general

#### Setting and model

This study describes a model study using the result of previous research on transition probability [7]. The study obtained the approval (E2536) of the Ethics Committee of Kyoto University. Written informed consent was obtained from the participants that were included in this study. The analysis had a public health care perspective, testing the optimal decision making for patients concerning health care services. At present in Japan, Implants and PFDPs are not insured medical examination options; accordingly, the study examined whether or not investment in Implants and PFDPs are more beneficial than IFDP when an individual patient thinks about investing in treatment for a prosthetic treatment for intermediate loss of molar. Hence, data was modelled using the Markov model for the assessment. TreeAge Pro 2015 (TreeAge Software Inc., Williamstown, MA, USA) was used for data modelling and analysis.

#### Targeted population

For the QOL parameters for the Markov model, a questionnaire survey was conducted to collect a QOL measurements. The subjects of the survey for the QOL measurements were male and female outpatients (*n* = 560) at the Department of Oral Implantology, Osaka Dental University, aged 37–81 years and who have lost the row of teeth in their lower jaw. The test subjects were classified into four groups, i.e., Implant treatment, FDP treatment, RPD treatment and those who were not treated for the loss.

#### Comparators

**Fig 1:**
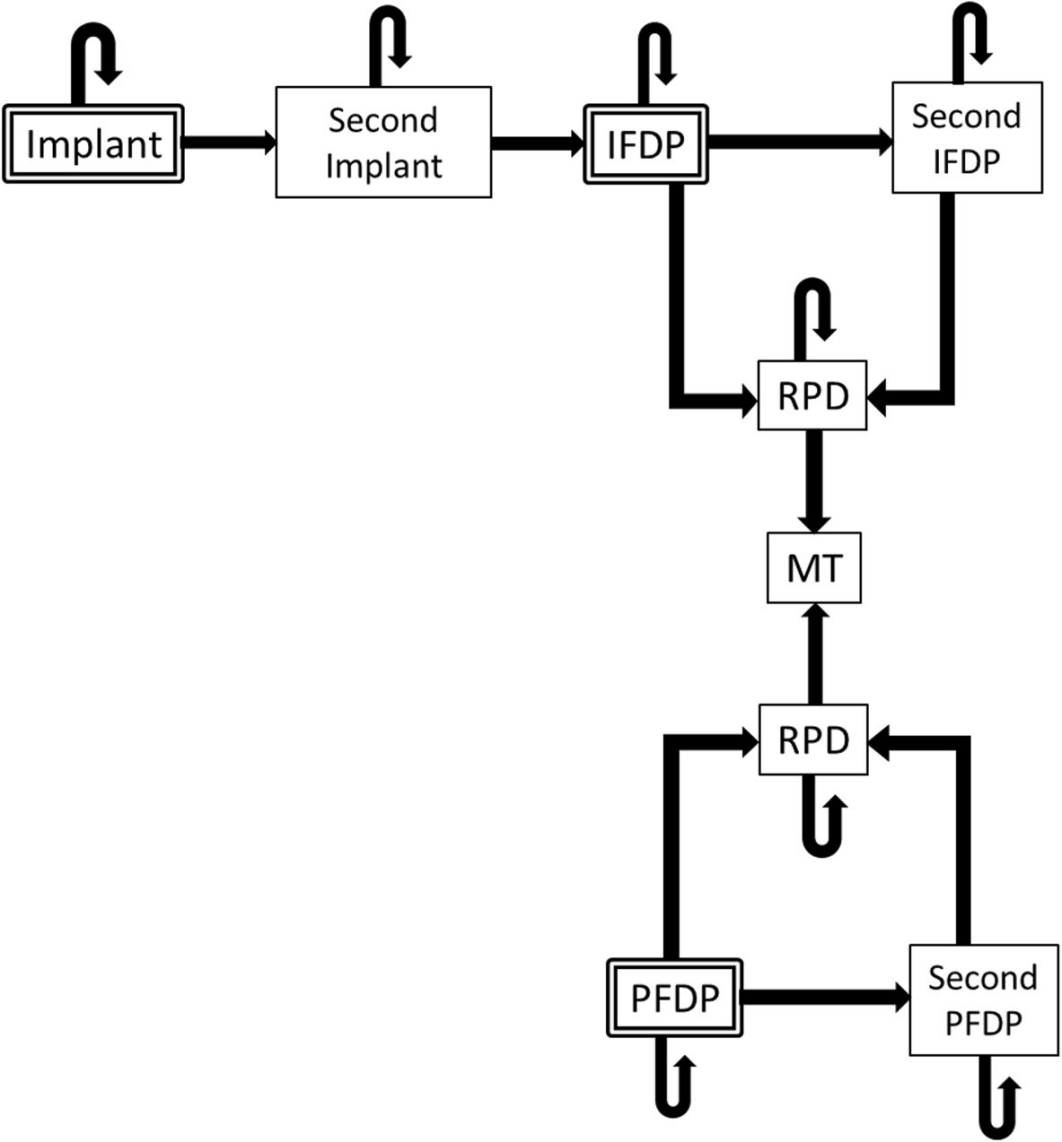
State transition diagram(:initial status)

Under this model, Implants, PFDPs and IFDPs were set as the decision node for prosthetic treatment of a missing unilateral lower jaw first molar. The model was created so that there would be transition to IFDP in the case of a lost Implant and transition to RPD where the IFDP failed. Regarding the transition probability of the implant, we do not include prosthetic failure and are based on the loss of the implant body. Where the RPD is not provided, the status was considered not treated (Fig. 1).

#### Time horizon

The mean number of missing teeth for persons aged 45–49 years in the Ministry of Health, Labour and Welfare’s Survey of Dental Diseases (2011) was 1.5 [8]. Consequently, under this model, 50 years was adopted as the age for the first prosthetic treatment for the loss of a molar. In addition, since Japanese average life expectancy exceeds 80 years for both men and women, the time horizon of the analysis was set at 30 years.

#### Discount rate

To adjust the future costs and health benefits to a present value at the decision making in terms of the time preference, a discount rate of 2% per year was considered in the analysis in accordance with Japanese guidelines [9].

### Outcome

#### Choice of outcomes

This study calculated the Incremental Cost-Effectiveness Ratio (ICER) with the CEA. In addition, the Cost-Effectiveness Acceptability Curves (CEAC) for Implant, IFDP and PFDP were plotted and probabilistic and deterministic sensitivity analyses conducted.

#### Measurement of effectiveness

The results of the questionnaire survey for the General Oral Health Assessment Index (GOHAI) [10], a comprehensive health-related QOL value that relates to the oral cavity, were used for measurements of the effect in this study. GOHAI comprises three areas (subordinate scales) that measure the extent of physical and psychosocial limits on aspects of living caused by oral difficulties. Eating, swallowing and pronunciation are assessed for physical function; aesthetic appreciation and sociability are assessed for psychosocial function. In addition, items such as relating to the use of medicines and hypersensitivity are assessed for pain and discomfort. A total score for 12 items from these three areas is assessed. GOHAI is calculated to be in the range of 12–60, but for this study the GOHAI value was converted to the continuous value between 0 and 1 based on beta distribution (0:no satisfaction, 1:full satisfaction); that is, a converted GOHAI value of 1 for the subjects indicates absolutely no limitations on physical and psychosocial aspects of living. Conversely, 0 indicates pronounced limitations on the physical and psychological aspects of living due to oral difficulties. The subjects of the survey comprised outpatients of the Department of Oral Implantology, Osaka Dental University Hospital between September 2014 and March 2016. In addition, a bias in the QOL due to missing teeth is expected when assessing the QOL value of the subjects. Thus, the target analysis under this study was the calculation of QOL value for each prosthetic device, with a separate classification of the missing teeth by the Kennedy Classification and the Eichner Classification systems. Furthermore, the accumulation of GOHAI was done after registering the application to use iHope International K.K.’s QOL scale. Expert opinion was used due to lack of previous research.

#### Modeled parameters

Data from previous research were used for transition probability and sourced from three systematic reviews [11–13] and three retrospective cohort studies [14–16]. In addition, annual mortality rates were calculated using the FY2013 Abridged Life Table from the life table published by the Statistics Bureau of the Ministry of Internal Affairs and Communications in Japan [17]. For one issue (the failure rate of renewed FDP treatment), the calculation was based only on expert opinion. The annual failure rate of each type of prosthetic device, the distribution and probability of treatment following failure, annual mortality rate and the parameters for the data source are presented in Table 1.

**Table 1:** Distributions of annual failure rates and allocation on several stages used in the model

State	Annual failure rate (%)	Allocation		Data source
		Allocated to	Prob.	
Implant	0.52	second Implant	1	Jung et al. (2012) [11] (systematic review)
second Implant	2	IFDP	1	Mardinger et al. (2012) [14] (retrospective)
IFDP	11	second IFDP	0.998	Aoyama et al. (2008) [15] (retrospective)
		RPD	0.002	Pjetursson et al. (2007) [12] (systermatic review)
second IFDP	15	RPD	1	assumption
PFDP	4.4	second PFDP	0.998	Torabinehad et al. (2007) [13] (systematic review)
		RPD	0.002	Pjetursson et al. (2007) [12] (systermatic review)
second PFDP	8.4	RPD	1	assumption
RPD	16.8	MT	1	Jepson et al. (1995) [16] (retrospective)
all state	50 years: 0.0016	dead	1	e-stat https://www.e-stat.go.jp/SG1/estat/GLO8020103.do?_toGLO8020103_&listID=000001120139&requestSender=dsearch
	51 years: 0.0017			
	:			
	80 years: 0.0252			

### Costs

Since the study was conducted in Japan, the calculating process of the cost parameters conformed to Japanese medical care insurance system. An exchange rate of €1 = ¥114.60 (as at 26 July 2016) was used [18], and gamma distribution was adopted as the distribution in the probabilistic sensitivity analysis for cost of each treatment. PFDP is not an insured procedure under the public medical care insurance system, and the fee for medical services is not fixed; therefore, reference was made to the general cost of treatment in Japan. For Implants, there have been allowances of some insured procedures in Japan (cases where jawbones have been lost or damaged extensively due to disease or accident). This study calculated the cost equivalent for one missing tooth referring to the applied medical service fee.

## Results

### Study parameters

Table 2 shows the results of the beta distribution of the GOHAI value used in this study converted to 0 and 1. The results for lost Implants and lost FDP are also reported. In this study, subjects who have undergone Implants with the Kennedy Classification of level 2 and Eichner Classifications of B1 and B2 (loss of tooth) are considered to be in the Implant group. This corresponds to 168 Implant patient with a QOL value of 0.88 ± 0.14. In addition, Table 3 illustrates the cost of prosthetic treatment and the post treatment maintenance cost.

**Table 2:** Patient’s satisfaction survey on several stages

State	Kennedy Classification	Eichner Classification	Distribution	No. of patients	Age Mean ± 1SD	Distribution parameters Mean ± 1SD
Implant			Beta	168	61.7 ± 9.8	0.88 ± 0.14
	II	B1-B2				
lost Implant			Beta	32	61.1 ± 9.4	0.71 ± 0.23
FDP			Beta	65	59.0 ± 11.4	0.83 ± 0.13
	III	A2-A3-B1-B2				
lost FDP			Beta	66	54.8 ± 11.5	0.68 ± 0.17
RPD	II-III	B1-B2	Beta	45	63.8 ± 10.3	0.71 ± 0.23
MT	II	B1-B2	Beta	184	59.1 ± 11.0	0.70 ± 0.18

**Table 3:** Cost survey on several stages. (€)

State	Distribution	Distibution Mean ± 1SD (€)	Data source
Implant	Gamma	2744 ± 274.4	Interpolated fromhealth insurance treatment costsof Japan
IFDP	Gamma	420 ± 42.0	Health insurance treatment costsof Japan
PFDP	Gamma	2618 ± 261.8	Private practice
Implant•FDP•MT maintenance	Gamma	261.8 ± 26.2	Interpolated from health insurance treatment costs of Japan
RPD	Gamma	368 ± 36.8	Health insurance treatement costs of Japan
RPD maintenance	Gamma	305 ± 43.6	Health insurance treatment costs of Japan

### Incremental costs and effectiveness

**Fig. 2:**
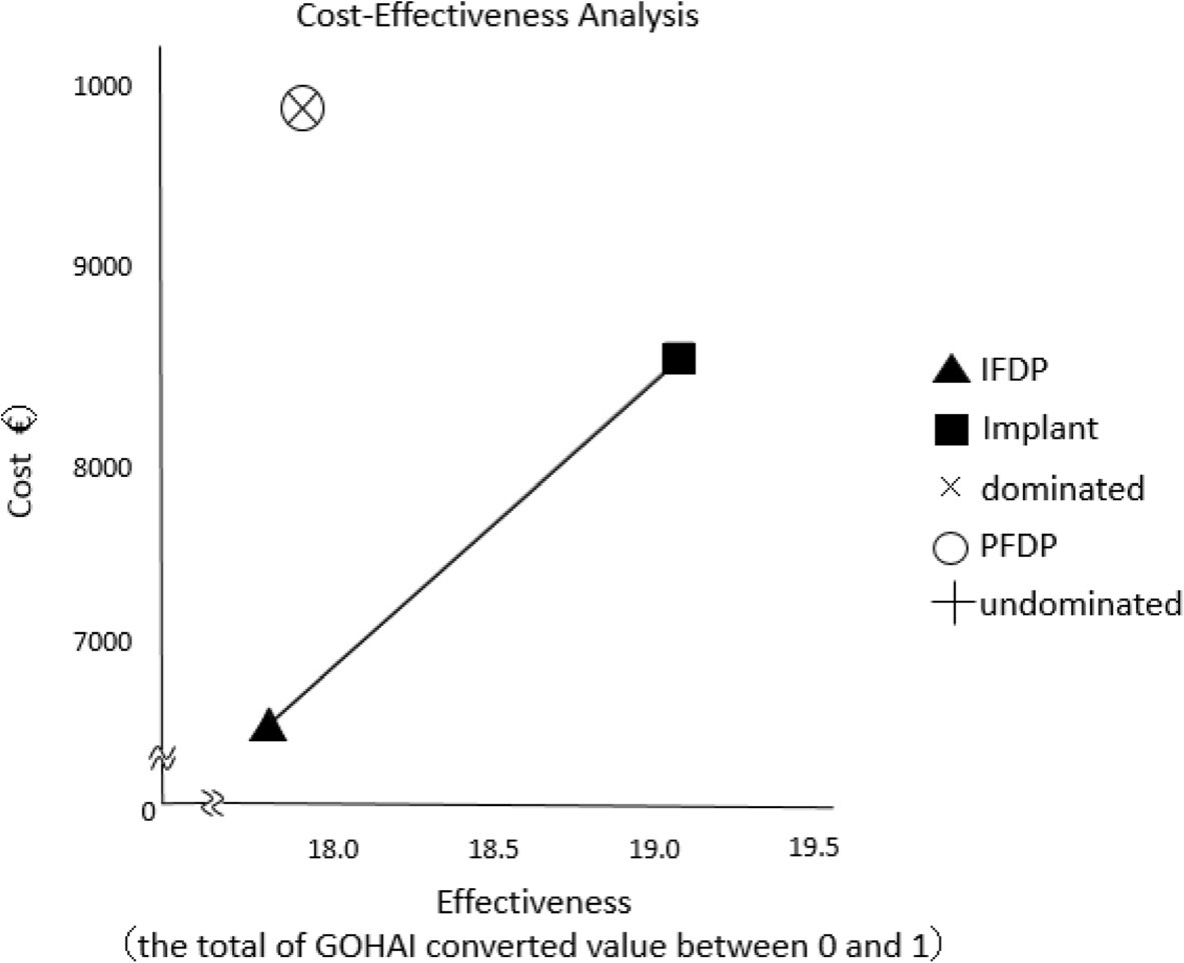
Results of cost-effectiveness analysis

The results plotted for the cost and effect of the three groups in the cost-effectiveness plane are shown in Fig. 2. PFDP is to the upper left of the direct line for IFDP and Implants, indicating it to be extended dominated. The results for the calculated ICER are shown in Table 4. The cost difference between Implant and IFDP is €1849.90, while the difference in effectiveness is 1.3; thus, the ICER was €1423.00.

**Table 4:** Incremental cost-effectiveness ratios of Implant versus IFDP

Category	Strategy	Cost	Incr cost	Eff	Incr eff	Incr C/E (ICER)
Excluding dominated						
Undominated	IFDP	6611.2		17.8		
Undominated	Implant	8461.1	1849.9	19.1	1.3	1423

### Characterizing uncertainty

**Fig. 3:**
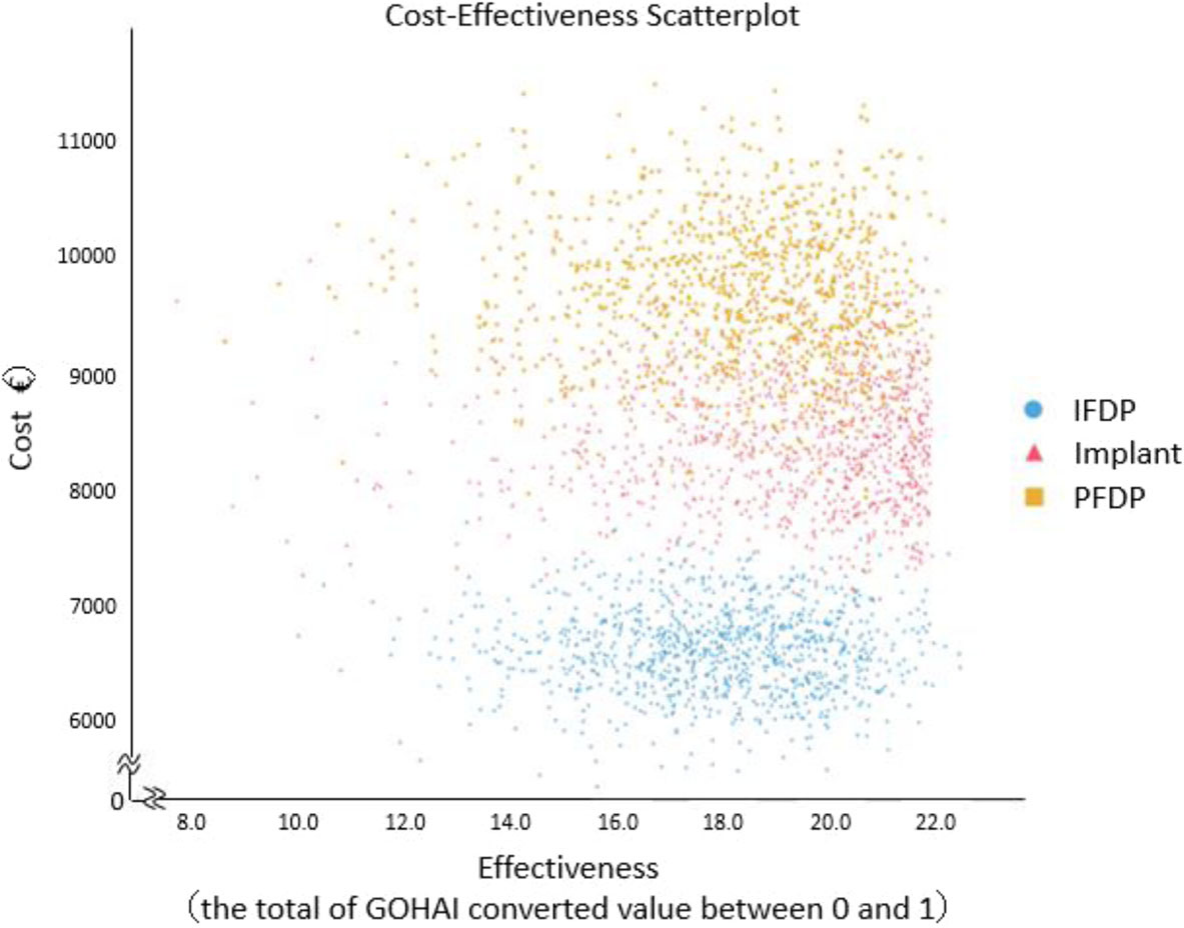
The cost-effectiveness plane (Monte-Carlo simulation)

**Fig. 4:**
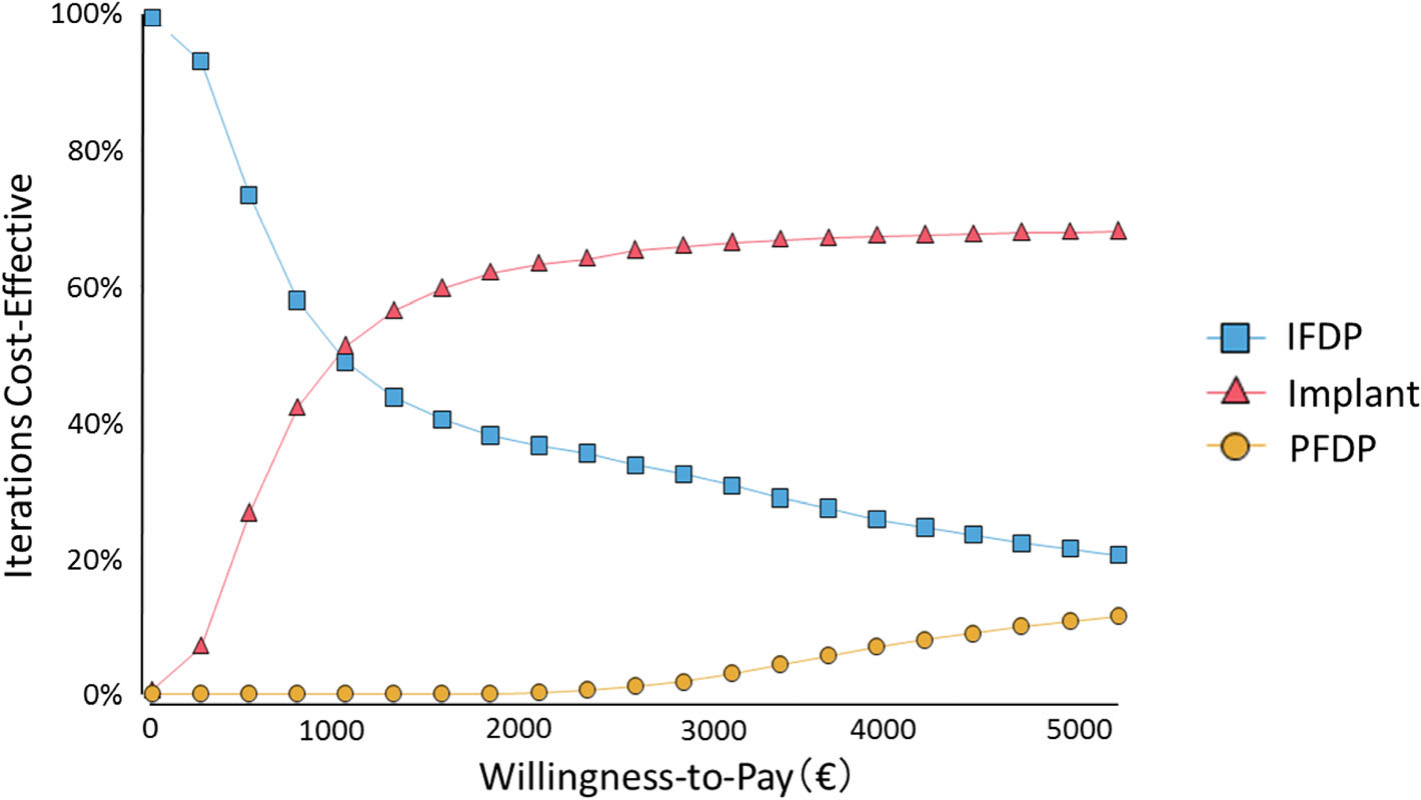
Cost-effectiveness acceptability curves

**Fig. 5:**
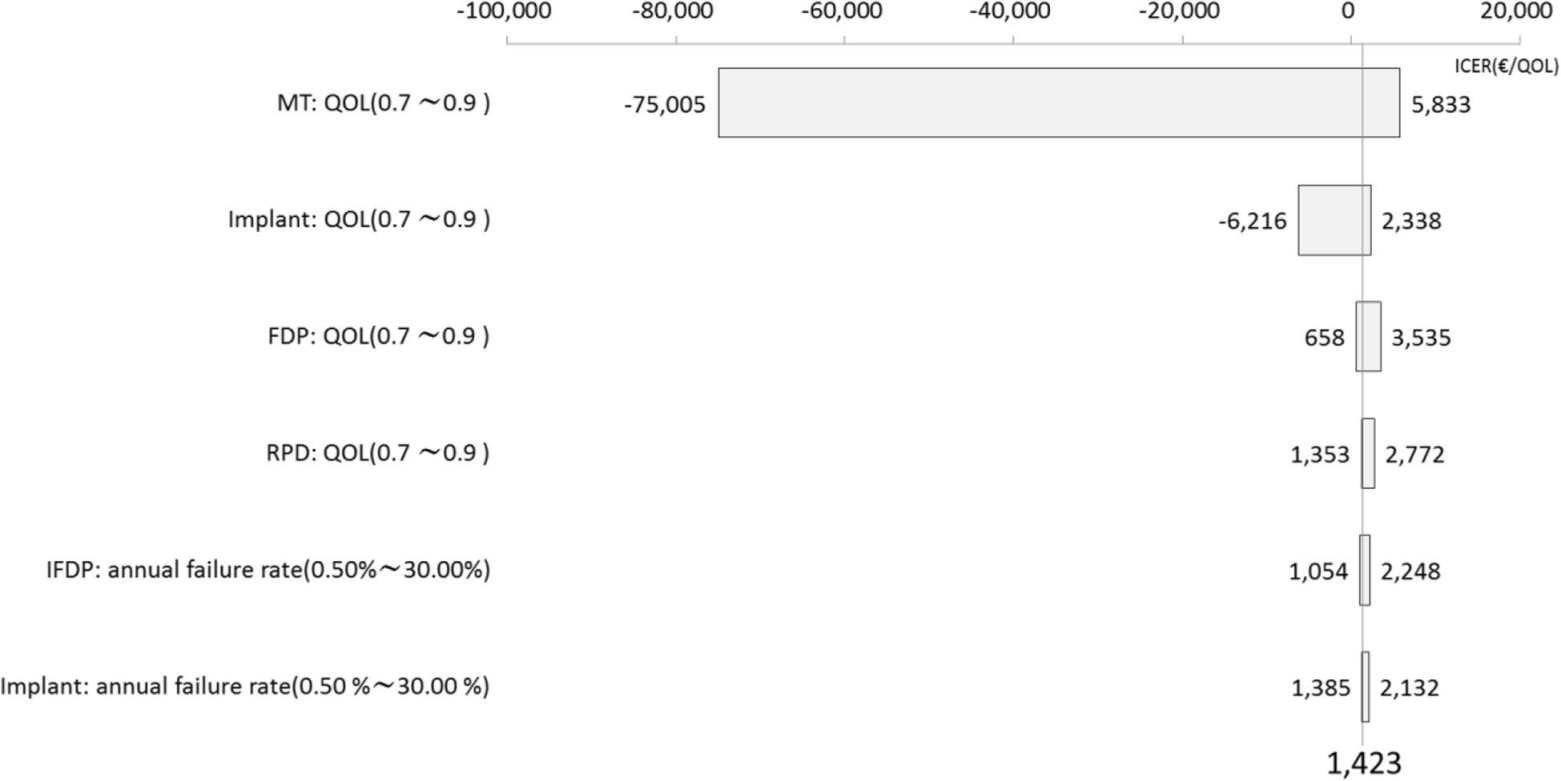
Sensitivity analysis comparing Implant and IFDP (Tornado diagram)

As a probabilistic sensitivity analysis, the results using Monte-Carlo simulations repeated 5000 times were plotted on the cost effectiveness plane (Fig. 3). The IFDP and PFDP points are widely distributed, but Implants are concentrated on the right-hand side of the plane compared with the other two groups. The CEAC is shown in Fig. 4. This illustrates how IFDP has a higher permissibility than other treatments where the willingness to pay (WTP) threshold is low. In addition, admissibility also commenced for PDFP at over €3000. The results of a deterministic sensitivity analysis are illustrated in Fig. 5. The results of 1-way sensitivity analysis shown in the Tornado diagram indicate the variable with the largest level of variation in the ICER to be the parameter of not treating the missing tooth, which indicates the high priority for additional research. In addition, the QOL value for Implant has a much larger impact on ICER than the Implant failure rate.

## Discussion

### Study finding

To the best of our knowledge, this study is the first health economic evaluation using Japanese medical care data (cost/QOL value) for prosthetic treatment of intermediate loss of a single tooth in the molar region. In addition, studies of cost-effectiveness in dentistry often take the survival period to be the effectiveness [7, 19]; however, this study assessed the effect from a QOL perspective. In previous research, cost effectiveness has been examined using independent questionnaires to measure QOL [20]; instead, this study used GOHAI, which is considered appropriate for measuring QOL from a suitability perspective. Such a result is considered useful not only from the perspective of the patient satisfaction level but also for designing insurance policies. Limitations of the study include that there was inevitable reliance on three retrospective cohort studies on treatment transition probabilities and that the re-application of the same prosthetic treatment following the failure of an Implant and FDP was restricted to only once. This research is focusing on a single intermediate missing tooth in the first molar, but the condition of the dentition in other parts is unknown. The state of the dentition of the other part is adjusted so as not to be biased by the Kennedy Classification and the Eichner Classification systems. However, the life and death of the pulp is unknown. Also we have chosen to not include IFDP as a transition from a failed PFDP. In addition, the QOL measurement used in this study is a potential limitation for the ability to generalise the results since the data source was from a single institution. Longitudinal data has to be collected from other institutions in future. The current situation for the medical field in Japan is that there are health institutions for the Diagnosis Procedure Combination (DPC) system and a documented database of fees for medical services for public health insurance and cooperative insurance. Observational studies that rival randomised controlled trials (RCTs) have been implemented by understanding and being reminded of the features of such large data [21, 22]. However, there is currently a lack of data being collected across other institutions in the area of dentistry, particularly in relation to Implants. In terms of the advancement of clinical research, construction of a database that permits identification and access to necessary information simply and rapidly is vital for developing the study of Implants.

### Effectiveness cost

According to the report from Naito et al., Japanese national norm for GOHAI is 52.2 ± 7.8 (0.84 ± 0.16 when converted to 0 to 1) for 50–59-year-olds [23]. Comparing with this result, the results of the current study can be considered the same as Implants and FDP that perform well over time. That is, lost Implants and removed FDP do not function as well as the national norms. In Japan, the medical care necessary to stay healthy and alive, such as for recovery from sickness and disability, delayed progress of sickness and disability, maintenance of physical and mental functions, is adapted from public medical care insurance according to the universal healthcare system. As a patient’s co-payment, patients aged 6–69 years pay 30% of medical care costs at reception while infants aged 0–5 years pay 20% (separate public subsidies provided by local governments) and elderly persons aged 70 and above pay 10% (or 30% depending on income). Unrelated to this, medical care for procedures such as cosmetic surgery, orthodontics and sex reassignment surgery are completely self-funded, and medical treatments are not covered by public insurance. IFDP in this study is adapted for public medical care insurance, whereas PFDP corresponds to self-funded medical treatment. Implants also generally correspond to self-funded medical treatment, but some are adapted for public medical care insurance; thus, the analysis refers to those cost data. The cost for Implants under self-funded medical treatment in Japan is approximately €2747.50–3927.50.

### Main result

In this study, starting with an Implant for the prosthetic treatment of a single missing tooth in the molar region of the lower jaw had a higher QOL condition than either IFDP or PFDP. However, IFDP had the lowest cost among the three groups when estimated for 30 years. In addition, it became clear that PFDP is an extended dominated state relative to IFDP and Implant. There was a trend for these results to resemble the cost effectiveness research for Implants and FDP conducted previously [6, 7, 20]. However, the method of analysis, including the one used in this study, has not been a cost–utility analysis (CUA); instead, all were cost-effectiveness analyses, and there was no consistency in the effectiveness index. Within the realm of medicine, EuroQOL-5Dimension (EQ-5D), Structured Form 6 Dimension (SF-6D) [24], Healthy Utility Index (HUI) [25] and other preference-base QOL questionnaire forms are used to calculate the Quality-Adjusted Life Year (QALY), which is an indicator used for CUA. However, in previous studies using such QOL forms, no calculations have been made for the detailed difference in the condition of the oral cavity. A report by Shiroiwa et al. confirmed a significant decline in the QOL value with SF-6D due to tooth disorder but did not confirm a significant difference with EQ-5D [24]. However, the detailed condition of the oral cavity was unknown in this result. In future, missing tooth treatments need to be assessed with particular focus on the different types.

## Conclusion

The study results suggest a better QOL can be obtained from an Implant than from IFDP or PFDP. However, the Tornado diagram in the deterministic sensitivity analysis suggests that additional research is required, particularly in relation to the QOL value for when a missing tooth is not treated. An evaluation form using an indexed scale for oral health related aspects needs to be developed that is also consistent as an indicator of effect. If this can be used to understand the extent to which the oral environment has an impact on the QOL by setting a standardised value for the state of oral health condition of citizens, then it could also achieve a CUA.

## Abbreviations

CEA: Cost effectiveness analysis
CEAC: Cost-Effectiveness Acceptability Curves
CUA: Cost–utility analysis
DPC: Diagnosis Procedure Combination
EQ-5D: EuroQOL-5Dimension
FDP: Fixed dental prosthesis
GOHAI: General Oral Health Assessment Index
HTAs: Health Technology Assessments
HUI: Healthy Utility Index
ICER: Incremental Cost-Effectiveness Ratio
IFDP: Insurance fixed dental prosthesis
PFDP: Private fixed dental prosthesis
QALY: Quality-Adjusted Life Year
QOL: Quality of life
RCTs: Randomised controlled trials
SF-6D: Structured Form 6 Dimension
WTP: Willingness to pay
